# Wenshenqianlie capsule improves benign prostatic hyperplasia via its anti-inflammatory and antioxidant effects

**DOI:** 10.18632/aging.206103

**Published:** 2024-09-04

**Authors:** Rui Liu, Zhen Sun, Shimiao Wang, Xin Liu, Yuhong Man, Meiwan Chen, Qian Liu, Chunyue Wang

**Affiliations:** 1 School of Life Sciences, Jilin University, Changchun 130012, China; 2 Engineering Research Center of Chinese Ministry of Education for Edible and Medicinal Fungi, School of Plant Protection, Jilin Agricultural University, Changchun 130118, China; 3 Center for Reproductive Medicine, Center for Prenatal Diagnosis, The First Hospital of Jilin University, Changchun 130021, China; 4 Department of Neurology, The Second Hospital of Jilin University, Changchun 130041, China; 5 State Key Laboratory of Quality Research in Chinese Medicine, Institute of Chinese Medical Sciences, University of Macau, Macau SAR 999078, China

**Keywords:** Wenshenqianlie, benign prostatic hyperplasia, anti-inflammatory, in vivo serum metabolomics, gut microbiomics

## Abstract

Anti-inflammatory and antioxidant effects play crucial roles in the recovery of benign prostatic hyperplasia (BPH). Wenshenqianlie (WSQL) capsule, a typical traditional Chinese medicine formulation combining 14 Chinese herbs, has been reported to exert tonic effects on the kidneys and improve clinical symptoms of BPH. However, its potential antioxidative and anti-inflammatory properties and effects on the improvement of hormone levels have not been reported in depth. In this study, mice were subcutaneously injected with TP (5 mg/kg·d^-1^) to induce BPH. Forty-eight adult BALB/c male mice were randomly allocated to six groups based on the type of drug administered by gavage: control, BPH, BPH+WSQL (40 and 80 mg/kg·d^-1^), BPH+finasteride (1 mg/kg·d^-1^), and WSQL-only treated (80 mg/kg·d^-1^). We investigated the anti-inflammatory and antioxidant effect and mechanism of WSQL on BPH via histopathological examination, immunohistochemistry, enzyme-linked immunosorbent assay, and western blotting combined with *in vivo* serum metabolomics, gut microbiomics analysis. WSQL alleviated prostate hyperplasia and reduced prostate-specific antigen, dihydrotestosterone, testosterone, and inflammation levels. Gut microbiomics and serum non-targeted metabolomics determined that the protective effect of WSQL against BPH may be related to the improvement of inflammation and testosterone-related gut microbiota and serum metabolites. Further studies showed that WSQL ameliorated nuclear factor-kappa B, its downstream inflammatory factors, and nuclear factor E2-related factor 2 pathway.

## INTRODUCTION

Benign prostatic hyperplasia (BPH) refers to a non-malignant prostate gland enlargement that is characterized by an abnormal proliferation of epithelial and stromal cells within the transition zone of the prostate [1], resulting in lower urinary tract symptoms [2]. Globally, BPH is associated with the highest total number of years lived with disability compared to other significant male genitourinary disorders, such as urolithiasis, bladder cancer, and prostate cancer [3], imposing a great burden on patients’ quality of life [4].

The mechanisms underlying BPH have not yet been completely elucidated; however, advanced age and functioning testes are major contributors [5]. Years of research into the etiology of BPH has led to the proposal of several hypotheses including hormonal-endocrine, cytokine, oxidative stress and inflammation, among others [6–8]. The hormonal-endocrine theory is the most widely discussed etiology of BPH. Dihydrotestosterone (DHT), the active metabolite of testosterone (T), is the most potent androgen in binding to androgen receptors in the male prostate, inducing prostate cell proliferation through growth factors [9]. Additionally, recent studies suggest that prostate inflammation is the primary reason that eventually leads to the further development of BPH. The inflamed prostate tissue undergoes a long-term cycle of damage and repair [10], potentially leading to constant stimulation of epithelial and stromal prostate cell proliferation. Interestingly, this may be related to an enhanced stimulatory effect of DHT on inflammatory factor secretion [11]. The inflammatory response can be exacerbated by the activation of nuclear factor-kappa B (NF-κB) following oxidative stress, and can be inhibited by antioxidant such as nuclear factor E2-related factor 2 (Nrf2) [12]. Recent studies have suggested that gut microbiota affects BPH, however, the precise underlying mechanisms remain elusive. Alterations in the gut microbiota can indirectly impact prostate health by activating the immune system and producing proinflammatory cytokines to improve chronic systemic inflammation [13]. The metabolites and gut microbiota can interact and thus influence disease progression [14–16]. Further, intestinal ecological dysregulation leads to episodes of BPH by altering metabolic profiles or affecting the levels of hormones such as T [17].

The current treatment options for BPH encompass surgical interventions and pharmacotherapy. The most frequently prescribed medications include 5α-reductase inhibitors (5-ARIs) and α1-blockers [18, 19]. Nevertheless, the utilization of 5-ARIs and α1-blockers is associated with multiple sexual and physical side effects, such as erectile dysfunction and cardiovascular risks [20–22], resulting in serious consequences in the vulnerable population affected by BPH, particularly middle-aged and older men [23, 24]. Additionally, the effectiveness of α1-blockers appears to diminish over time [19]. Therefore, developing new, effective, and safer therapeutic strategies for BPH is essential.

Wenshenqianlie (WSQL) capsule, a product derived from extensive clinical experience, consists of 14 Chinese herbal ingredients, including Epimedii Folium (dried leaves of *Epimedium brevicornu* Maxim. [Berberidaceae]), Rehmanniae Radix Praeparata (dried rhizome of *Rehmannia glutinosa* Libosch. [Scrophulariaceae]), Dioscoreae Rhizoma (dried rhizome of *Dioscorea opposita* Thunb. [Dioscoreaceae]), Poria (dried sclerotium of *Poria cocos* [Schw.] Wolf [Polyporaceae]), and Fructus Corni (dried fruit pulp of *Cornus officinalis* Sieb. et Zucc. [Cornaceae]). According to traditional Chinese medicine researchers, BPH causes kidney deficiency and dampness. WSQL has multiple beneficial effects and can treat BPH symptomatically by inhibiting the process and causing the symptoms mentioned above. The latest research has demonstrated that Epimedii Folium and its constituent icariin possess anti-inflammatory and antioxidant properties [25, 26], and can effectively treat BPH in rats [27]. Rehmanniae Radix Praeparata, Dioscoreae Rhizoma and Poria have several pharmacological effects such as antioxidant activity [28, 29], among which Poria polysaccharides mitigate oxidative stress, regulate hormone production, modulate the gut microbiota, and remodel DNA methylation, reducing in prostate weight and prostate index [30]. Fructus Corni administration attenuates T-induced BPH by suppressing 5α-reductase and androgen receptor expression [31]. Based on the cumulative clinical experience, WSQL innovatively integrates the efficacy of each ingredient and treats BPH symptomatically by tonifying the kidney and preventing dampness. However, there is a lack of reported studies investigating the mitigating effects of WSQL on BPH through its anti-inflammatory and antioxidant properties, as well as the underlying potential mechanisms.

In this study, we established a model of BPH in BALB/c mice by treating them with testosterone propionate (TP). We conducted a systematic study on the therapeutic effects of WSQL on BPH *in vivo*, and elucidated the underlying mechanism through histopathology, 16S rRNA gene sequencing and analysis, non-targeted metabolomic analysis, enzyme-linked immunosorbent assay (ELISA), and western blotting.

## MATERIALS AND METHODS

### Animal experimental protocols

All animal experiments were conducted under the supervision of the Jilin University Institutional Animal Care and Use Committee and following the guidelines of the Animal Ethics Committee of Jilin University (permit number: SY202111014). Male BALB/c mice aged 6 to 8 weeks were purchased from Liaoning Changsheng Biotechnology Co., Ltd. (SCXK (Liao) 2020-0001, Liaoning, China). Thirty-two mice were randomly divided into 4 groups (*n* = 8) and received a daily subcutaneous injection of 5 mg/kg TP (T101368, Shanghai Aladdin Biochemical Technology, Shanghai, China) dissolved in olive oil for BPH development for continued 4 weeks. At the same time, mice were intragastrically administered 10 mL/kg of isotonic sodium chloride solution, serving as BPH group (*n* = 8), 1 mg/kg of finasteride (FIN; F156753, Shanghai Aladdin Biochemical Technology, Shanghai, China) serving as BPH+FIN group, and daily doses of WSQL at 40 mg/kg and 80 mg/kg serving as BPH+WSQL groups for continued 5 weeks. Another 16 mice were injected with olive oil for continued 4 weeks, and orally received with 10 mL/kg of isotonic sodium chloride solution, serving as the control (Ctrl) group (*n* = 8), while the WSQL-only treated group (*n* = 8) received 80 mg/kg of WSQL. All mice were sacrificed after the last dose, euthanized by carbon dioxide inhalation (at 10%-20% of the chamber volume flow rate). Blood was collected from the tail vein. Fecal samples, and organs (prostate, liver, spleen, kidney, and heart) were collected and stored at −80° C, and samples were prepared for other analyses. All prostate lobes were collected for pathological and further biochemical analyses [32–34].

### Hematoxylin and eosin (H&E) staining

H&E staining was performed as previously described [35]. The excised prostate, heart, liver, spleen and kidney tissues were fixed, dehydrated, embedded and sectioned. Sections of these tissues were stained with H&E. The slides were observed using a light microscope (IX73, Olympus Co., Tokyo, Japan) and images were acquired to detect morphological changes in the tissues.

### Immunohistochemistry (IHC)

According to standard histological procedures, paraffin-embedded sections were used to assess prostate-specific antigen (PSA) expression in mouse prostate tissue. The embedded wax block of prostate tissue was sectioned for IHC. Antigen repair was conducted using citric acid (pH 6.0) antigen repair buffer (B2010, Wuhan Baiqiandu Biotechnology Co., Ltd, Wuhan, China). Prostate sections were incubated in 3% bovine serum albumin (BSA) for 30 min at 37° C and then incubated overnight at 4° C with a rabbit polyclonal antibody to PSA (1:100, af0246, Affinity Biosciences, Cincinnati, OH, USA). After washing with phosphate buffered saline (pH 7.4), the sections were incubated with horseradish peroxidase (HRP)-conjugated secondary Abs against an anti-rabbit IgG (5220-0336; SeraCare Life Sciences, Milford, MA, USA) at a dilution of 1: 1000 for 50 min at 37° C. The sections were washed again with phosphate buffered saline (pH 7.4), followed by diaminobenzidine coloration and hematoxylin re-staining. The IHC staining for PSA was interpreted after dehydration and blockade and further analyzed using ImageJ software (National Institutes of Health, Bethesda, Rockville, MD, USA).

### 16S rRNA gene sequencing and analysis

Fresh fecal samples from the Ctrl, BPH, and BPH+WSQL (80 mg/kg) groups were collected (*n* = 3) and stored at −80° C. Following the manufacturer’s protocols, the microbial genomic DNA was isolated from mouse fecal samples using the OMEGA Soil DNA Kit (D5625-01, OmegaBio-Tek, Norcross, GA, USA). The V3 and V4 regions of the 16S rRNA gene were amplified using polymerase chain reaction as in our previous study [36] and sequenced at Shanghai Personalbio Technology Co., Ltd. (Shanghai, China).

Paired-end sequencing of the community DNA fragments was performed on an Illumina platform (San Diego, CA, USA) by Personalbio Technology Co., Ltd. (Shanghai, China). The abundance tables of these sequences in the samples were called feature tables (corresponding to amplicon sequence variant [ASV] tables). The ASV abundance data from all samples were used to count the members in the Ctrl, BPH, and BPH+WSQL groups according to the grouping of samples, calculate the relationship between the three groups, and draw an ASV Venn diagram. For alpha diversity analysis, sequence data analysis was performed using QIIME2 (2019.4), and the indexes of Chao1, Observed_species, Shannon, Simpson, Faith_pd, Goods_coverage, and Pielou_e were calculated. We performed principal coordinate analysis (PCoA) using R scripts after pumping the ASV table flat to analyze beta diversity. The group means of relative abundance for each sample at the phylum level were visualized and plotted as a stacked bar chart to describe the compositional characteristics of each group. Species composition analysis was performed using a heatmap to compare the differences between the groups. Linear discriminant analysis (LDA) with effect size analysis was performed to generate a histogram of the distribution of LDA values from phylum to genus. We used the metagenomeSeq method to derive a metabolic pathway difference analysis graph to compare the BPH and BPH+WSQL groups.

### Serum metabolomic analysis

Serum samples (210 μL) (*n* = 3) were collected and made into samples to be separated and analyzed by ultra-high performance liquid chromatography-tandem mass spectrometry to obtain the raw data by Personalbio Technology Co., Ltd. (Shanghai, China), as previously described. Raw data were converted and subsequently imported into the freely available XCMS software to perform peak alignment, retain time correction, and extract the peak area.

After total peak intensity normalization, the processed data, such as those from orthogonal partial least squares discriminant analysis (OPLS-DA), were analyzed. Variable importance for the projection (VIP) was calculated for each variable in the OPLS-DA model to predict biologically significant differential metabolic molecules. Student’s *t*-test at the univariate level was further used for metabolites with VIP > 1 to measure the significance of each metabolite, and *p* < 0.05 was considered significant. Significantly different metabolites were selected to draw the Kyoto Encyclopedia of Genes and Genomes (KEGG) enrichment pathway diagram (bubble diagram) and a heatmap using the GraphPad Prism 8 software (Boston, MA, USA). Potential biomarkers from non-targeted metabolomics and potential differential gut microbiota from fecal 16S rRNA gene sequencing and analysis were combined to generate an association heatmap.

### Enzyme-linked immunosorbent assay

Serum and prostate samples were stored at − 80° C for short-term preservation until analysis. The serum samples were diluted according to the instructions. Prostate samples obtained from each group were ground at 60 Hz in 200 μL of saline twice for 30 s, using a high-throughput tissue grinder (Scientz-48; Scientz Biotechnology, Ningbo, China), and the supernatant was aspirated. The supernatant was collected by centrifugation (3500 rpm, 10 min) using a centrifuge (Allegra 64R; Beckman Coulter, CA, USA) at 4° C for the subsequent bicinchoninic acid assay (23225; Thermo Fisher Scientific, Waltham, MA, USA) and ELISA. The levels of T (ml001948), androstenedione (ASD, a precursor substance of T, ml001954), interleukin (IL)-1β (ml301814), IL-6 (ml063159), and transforming growth factor (TGF)-α (ml001867) in the serum, as well as the concentration of catalase (CAT, ml037752) and superoxide dismutase (SOD, ml643059) in the prostate of BALB/c mice, were determined according to the manufacturer’s instructions provided in commercially available ELISA kits purchased from Shanghai Enzyme-linked Biotechnology Co., Ltd. (Shanghai, China).

### Western blotting

The total protein from the prostate tissue was lysed using a protease and phosphatase inhibitor cocktail (P002, NCM Biotech, Suzhou, China) and radioimmunoprecipitation assay lysis buffer (20-188, Millipore-Sigma, Burlington, MA, USA). After separation of 40 μg of total prostate protein using electrophoresis with a 10% one-step polyacrylamide gel electrophoresis gel fast preparation Kit (PG212, Shanghai Epizyme Biomedical Technology, Shanghai, China), the protein was transferred to 0.45 μm polyvinylidene difluoride membranes (10600023, Cytiva, Marlborough, MA, USA), which were then blocked with a high-performance closure solution (GF1815, Shanghai Genefist, Shanghai, China). The membranes were incubated overnight with different primary antibodies, including Nrf2, SOD-1, heme oxygenase-1 (HO-1), CAT, IL-1β, IL-6, phosphor (p)-NF-κB, total (t)-NF-κB, tumor necrosis factor (TNF)-α, and glyceraldehyde-3-phosphate dehydrogenase (GAPDH). After washing, the membranes were incubated with secondary antibodies (goat anti-mouse or goat anti-rabbit) for 4 h at 4° C. Antibodies used for western blotting are listed in Table 1. Protein expression intensity was detected using an electrochemiluminescence detection kit (GK10008, GLPBIO, Montclair, CA, USA), and protein levels were further analyzed using an automated chemiluminescence image analysis system (Tanon 5200; Tanon Science & Technology, Shanghai, China) and the ImageJ software (National Institutes of Health, Bethesda, Rockville, MD, USA). Densitometry was used to quantify the protein levels and the bands were normalized to GAPDH expression, except for the p-NF-κB band, which was normalized to t-NF-κB expression.

**Table 1:** Antibodies used for western blotting.

**Antibody**	**Molecular weight**	**Catalog number**	**Dilution**	**Company**	**Area**
Nrf2	110 kDa	A1244	1:2000	ABclonal	Wuhan, China
SOD-1	16 kDa	A12537	1:2000	ABclonal	Wuhan, China
HO-1	33 kDa	A19062	1:2000	ABclonal	Wuhan, China
CAT	60 kDa	A11220	1:2000	ABclonal	Wuhan, China
IL-1β	31 kDa	A16288	1:2000	ABclonal	Wuhan, China
IL-6	23 kDa	ab214426	1:2000	abcam	Cambridge, MA, USA
p-NF-kB	65 kDa	AF2006	1:2000	Affinity Biosciences	Cincinnati, OH, USA
t-NF-kB	65 kDa	66535-1-Ig	1:2000	Proteintech	Chicago, IL, USA
TNF-α	26 kDa	17590-1-AP	1:2000	Proteintech	Chicago, IL, USA
GAPDH	36 kDa	E-AB-48017	1:2000	Elabscience	Wuhan, China
Goat Anti-Rabbit IgG-HRP	/	E-AB-1003	1:2000	Elabscience	Wuhan, China
Goat Anti-Mouse IgG-HRP	/	AS003	1:2000	ABclonal	Wuhan, China

### Statistical analysis

All data are expressed as the mean ± standard error of the mean (SEM). Statistical analysis was conducted with a one-way analysis of variance with Tukey’s post hoc test, except for the analysis of weight, for which a two-way analysis of variance with Dunnett’s multiple comparisons test was applied using DSS 25.0 (IBM, Armonk, New York, NY, USA). Statistical significance was set at *p* < 0.05.

### Data availability statement

The authors confirm that the data supporting the findings of this study can be found in the article. The datasets presented in this study can be found in online repositories. The names of the repository/repositories and accession number(s) can be found below: https://www.ncbi.nlm.nih.gov/bioproject/PRJNA947775.

## RESULTS

### WSQL alleviated prostate enlargement and reduced TP-induced elevation in DHT, T, ASD, TGF-α in serum and PSA level in prostate

**Figure 1:**
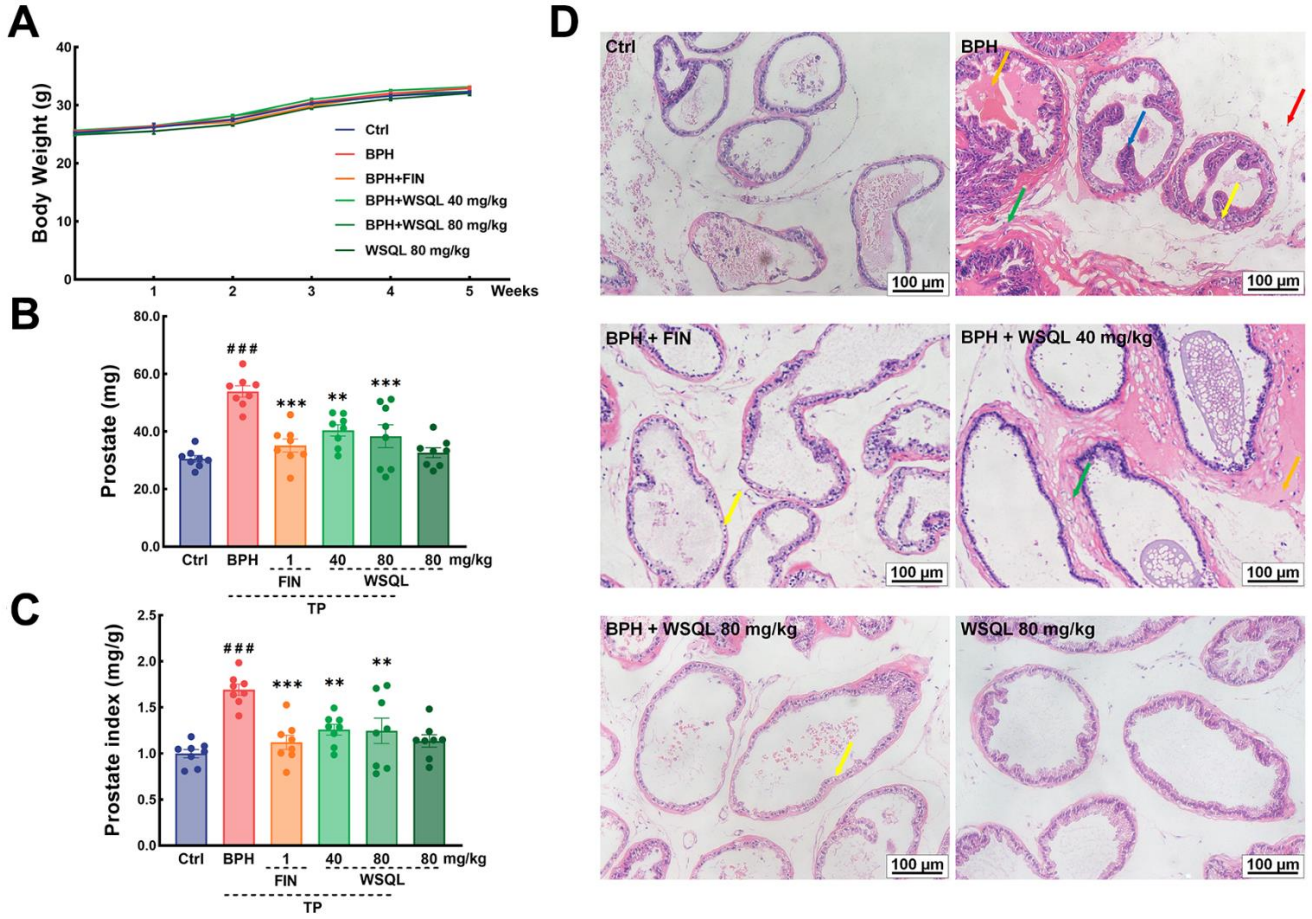
**Effect of WSQL on prostate injury and hyperplasia in TP-induced BPH model mice.** (**A**) Body weight, (**B**) prostate weight and (**C**) prostate index changed in mice during the experiment. (**D**) H&E staining of prostate tissues. Yellow arrow: epithelial cell degeneration; Green arrow: hyperplasia of connective tissue with tight arrangement; Orange arrow: eosinophilic exudate; Red arrow: inflammatory cell infiltration; Blue arrow: epithelial cell hyperplasia with papillary projection. Magnification: 200×, scale bar: 100 μm. ^###^ *p* < 0.001 vs. Ctrl group, *** *p* < 0.001 and ** *p* < 0.01 vs. BPH group. *n* = 8 for (**A**–**C**) and *n* = 3 for (**D**).

**Figure 2:**
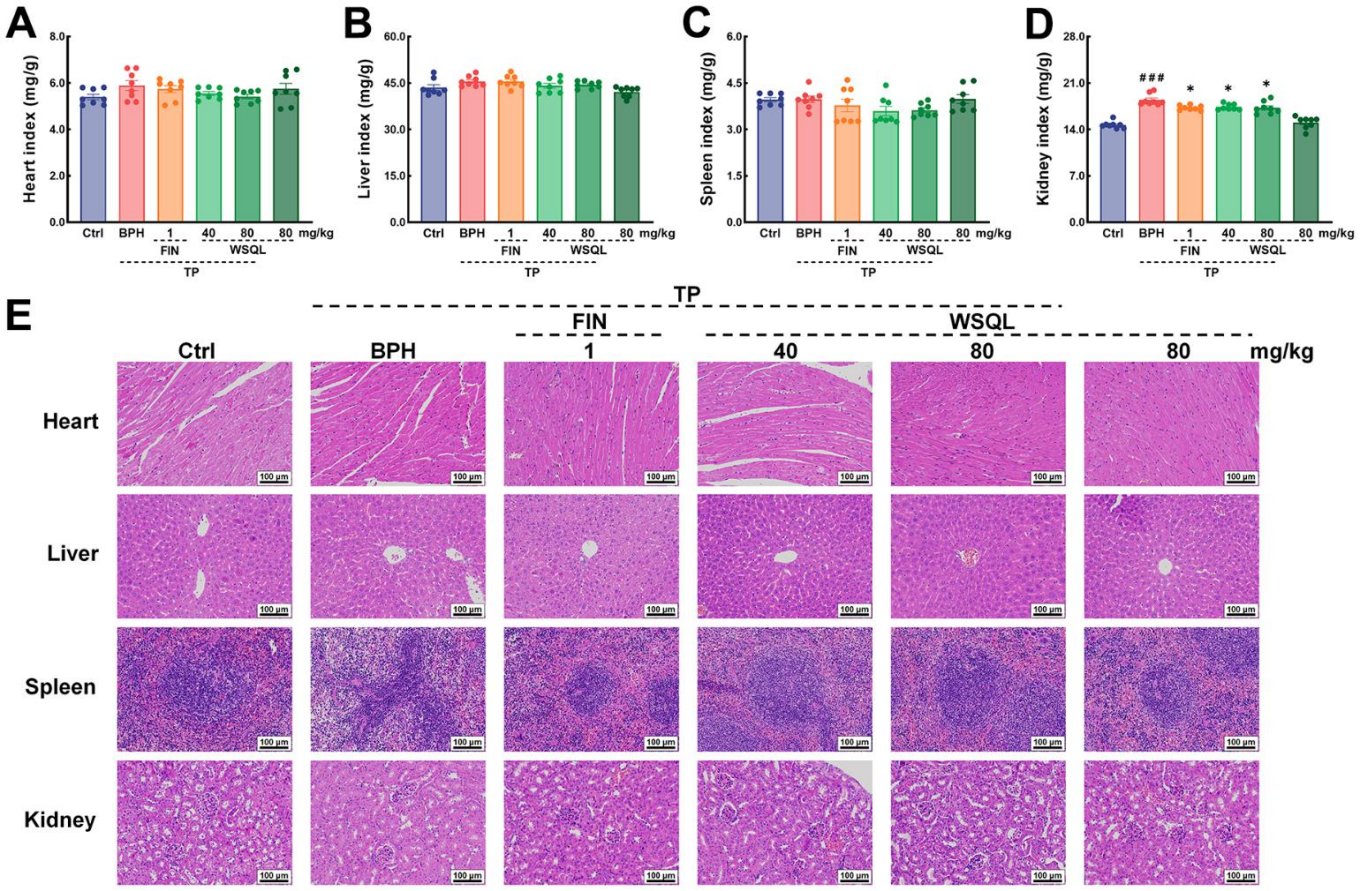
***In vivo* security of WSQL.** The indexes of (**A**) heart, (**B**) liver, (**C**) spleen and (**D**) kidney. (**E**) H&E staining of heart, liver, spleen and kidney. Magnification: 200 ×, scale bar: 100 μm. The data are presented as the mean ± SEM. ^###^ *p* < 0.001 vs. Ctrl group, * *p* < 0.05 vs. BPH group. *n* = 8 for (**A**–**D**) and *n* = 3 for (**E**).

**Figure 3:**
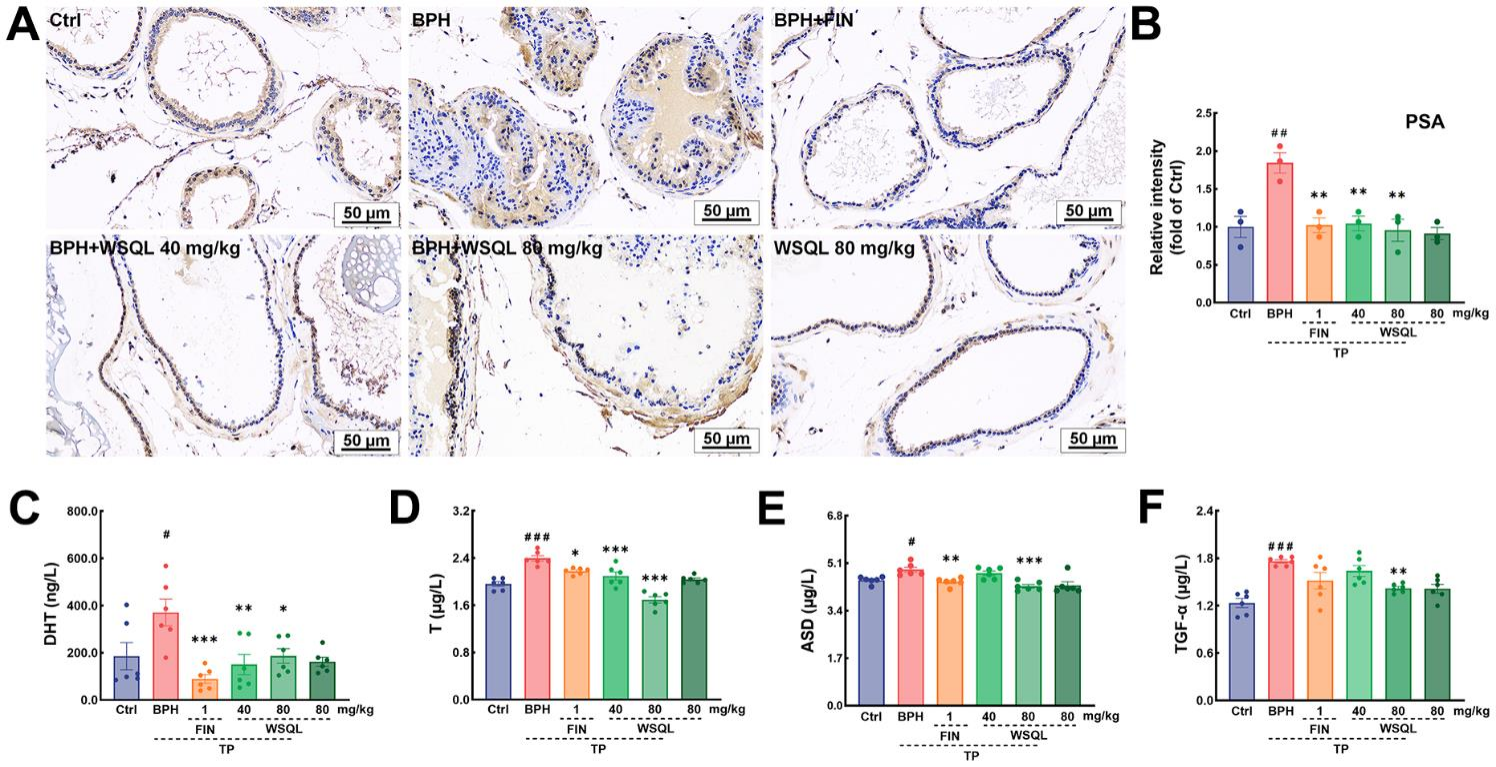
**WSQL improved BPH symptoms and reduced serum hormone and growth factor levels.** (**A**) IHC staining for PSA in prostate. Magnification: 200×, scale bar: 50 μm. (**B**) Quantitative analyses of IHC staining for PSA in prostate were reported as the fold change of the Ctrl group. Effect of WSQL on levels of (**C**) DHT, (**D**) T, (**E**) ASD and (**F**) TGF-α in serum. The data are presented as the mean ± SEM. ^###^ *p* < 0.001, ^##^ *p* < 0.01 and ^#^ *p* < 0.05 vs. Ctrl group, *** *p* < 0.001, ** *p* < 0.01 and * *p* < 0.05 vs. BPH group. *n* = 3 for (**A**) and (**B**) and *n* = 6 for (**C**–**F**).

To evaluate the safety and efficacy of WSQL on BPH, we measured daily changes in mouse body weight, examined organ indexes, and conducted histological staining upon completion of the experiment. TP modeling or FIN and WSQL administration did not significantly affect the body weight or indexes and histological staining of organs including the heart, liver, and spleen of the mice (Figures 1A, 2A–2C, 2E), thus indicating the safety of using WSQL. However, the kidney index in the BPH group was significantly higher compared to that in the Ctrl group (*p* < 0.001) (Figure 2D); however, it improved after FIN and WSQL administration (*p* < 0.05), which reflected the effect of BPH on the urinary system. Furthermore, compared to that in the BPH group, FIN and WSQL administration significantly decreased prostate weight and index (*p* < 0.01; Figure 1B, 1C). Histopathological analysis of prostate sections (Figure 1D) showed tubular glands with thin-walled vesicles in the Ctrl group. FIN and WSQL treatment ameliorated the papillary proliferation of epithelial cells protruding into the glandular lumen (blue arrow), hyperplasia of densely arranged connective tissue visible in the interstitium (green arrows), and infiltration of inflammatory cells (red arrow) that appeared after TP injection. Sparse epithelial cell cytoplasm with vacuolization (yellow arrows) was still present in the BPH+FIN and BPH+WSQL (80 mg/kg) groups. Mesenchymal hyperplasia (green arrows) and eosinophilic secretions (orange arrows) were still observed in the BPH+WSQL (40 mg/kg) group. PSA is a marker of the prostate and is significantly enriched in BPH. Severe prostate inflammation can elevate PSA levels [37]. The therapeutic effect of WSQL on BPH was evaluated by performing IHC staining for PSA in the prostate. PSA expression was observed in all groups and was significantly lower in the BPH+FIN and BPH+WSQL groups compared to that in the BPH group (*p* < 0.01) (Figure 3A, 3B). With these results, we conclude that WSQL is an effective and safe treatment option for BPH, with minimal impact on the body weight and organs of the mice.

The prostate is an androgen-dependent organ. DHT, an androgen derived from T through the action of 5α-reductase enriched in the prostate, is the most active form of androgen in the prostate and is an acute mediator of BPH [38]. Androgens such as DHT and T are significantly elevated after BPH modeling via TP injection [34, 39]. ASD is the substances that is able to synthesize T and DHT respectively in two androgen synthesis pathways [40]. ASD, a T precursor, can be converted to DHT at deficient levels, thus complementing the stimulatory effects of BPH [41]. To determine the effect of WSQL on serum hormone characterization, we measured the levels of DHT, T, and ASD using ELISA kits. After TP subcutaneous injection, the serum levels of DHT and T were significantly higher (*p* < 0.05) compared to those in the Ctrl group, while they significantly decreased upon FIN and WSQL administration (*p* < 0.05) (Figure 3C, 3D). Moreover, the level of ASD was significantly higher in the BPH group compared to that in the Ctrl group (*p* < 0.05) (Figure 3E) and was lower after FIN and WSQL (80 mg/kg) administration (*p* < 0.01).

TGF-α can stimulate prostate growth, and its expression is believed to be upregulated by T [42]. WSQL (80 mg/kg) administration reversed the increase in serum TGF-α levels observed after TP injection (*p* < 0.01, Figure 3F).

### Effects of WSQL on the gut microbiota

**Figure 4:**
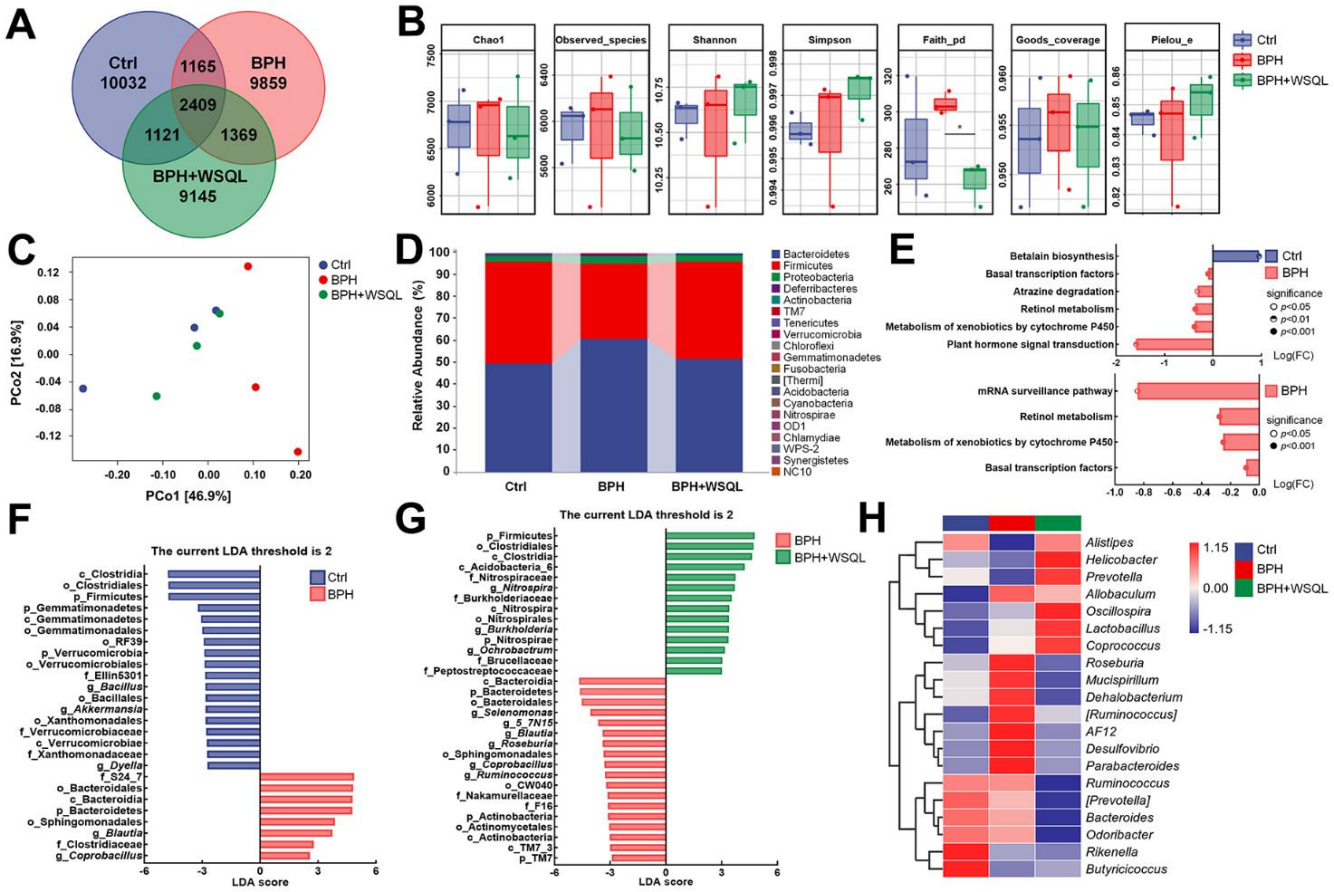
**WSQL treatment altered the abundance and diversity of the gut microbiota in BPH of BALB/c mice.** (**A**) ASV Venn diagram. (**B**) Alpha diversity according to Chao1, Observed_species, Shannon, Simpson, Faith_pd, Goods_coverage and Pielou_e. (**C**) Beta diversity according to PCoA analysis of a weighted_unifrac distance matrix. (**D**) The taxonomic distributions and relative abundance (%) of top 20 gut microbiota from each group at phylum level. (**E**) Differential metabolic pathways associated with differential gut microbiota between Ctrl and BPH groups and BPH+WSQL and BPH groups. LDA of differential taxa between (**F**) BPH and Ctrl groups and (**G**) BPH+WSQL and BPH groups according to the preset value (LDA > 2, *p* < 0.05). (**H**) Heatmap of top 20 gut microbiota at genus level. Taxa names are abbreviated as “p”, phylum, “c”, class, “o”, order; “f”, family, and “g”, genus. * *p* < 0.05 vs. BPH group. *n* = 3 for (**A**–**H**).

As the development of BPH is associated with alterations in the gut microbiota [17], we performed 16S rRNA gene sequencing to verify the function of WSQL in the gut microbiota. The Venn diagram demonstrated that out of the 35,100 ASVs identified in the rare pyrophosphate sequencing dataset, 2,409 ASVs were detected in all three groups. In contrast, the numbers of ASVs specific to Ctrl, BPH, and BPH+WSQL groups were 10,032, 9,859, and 9,145, respectively (Figure 4A). The Chao1, Observed_species, Shannon, Simpson, Goods_coverage, and Pielou_e indexes reflected low within-habitat diversity among the three groups. However, the Faith_pd index (*p* < 0.05, Figure 4B) showed higher diversity in the BPH group than in the WSQL group. PCoA revealed a large inter-individual component variation based on the weighted_unifrac distance matrix (Figure 4C). The Ctrl, BPH, and BPH+WSQL groups were well separated, with the BPH+WSQL group showing a closer projection distance to the Ctrl group, indicating a higher similarity in their community compositions. The taxonomic composition analysis at the phylum level revealed that Bacteroidetes (51.13%) and Firmicutes (44.30%) were the two dominant phyla among all taxa in the BPH+WSQL group, followed by Proteobacteria (3.04%), Deferribacterota (0.46%), and Actinobacteria (0.15%) (Figure 4D). Subsequently, we predicted the functional potential of the microbiota to identify metabolic pathways that may be associated with differential microbiota. A comparison of BPH with the Ctrl and BPH+WSQL groups (Figure 4E) revealed the following metabolic pathways to be enriched in the BPH group: basal transcription factors, retinol metabolism, and metabolism of xenobiotics by cytochrome P450 (CYP450) (*p* < 0.001). LDA comparing the BPH group with the other two groups (Figure 4F, 4G) revealed that at an LDA threshold of two, the marker species (biomarkers) of the BPH group were p_Bacteroidetes, g_*Blautia*, g_*Coprobacillus*, o_Bacteroidales, o_Sphingomonadales, and c_Bacteroidia, and the common biomarkers of the Ctrl and BPH+WSQL groups were p_Firmicutes, o_Clostridiales, and c_Clostridia. To further determine the changes in the microbial communities in the cecum of the three groups, 20 genera and their relative abundance were depicted using a heatmap (Figure 4H and Table 2). The species clustering algorithm, on average, revealed an increase in the relative abundance of the genera *Alistipes* and *Butyricicoccus* in the BPH+WSQL group compared with that in the BPH group and a decrease in the relative abundance of the genera *AF12*, *Desulfovibrio*, *Parabacteroides*, and *Allobaculum*, indicating that the gut microbiota could be associated with the effect of WSQL treatment during BPH progression. The results confirmed that WSQL increased the abundance of beneficial bacteria, such as *Prevotella*, and decreased the abundance *Blautia* [43, 44], thus altering the composition of the intestinal microbiota in BPH mice and involving certain metabolic pathways that may be related to hormone metabolism.

**Table 2:** Relative abundance of top 20 genera (*n* = 3).

**Sample**	**Ctrl**	**BPH**	**BPH+WSQL**
*Oscillospira*	0.03481	0.03635	0.04099
*Bacteroides*	0.02826	0.02738	0.02323
*Lactobacillus*	0.01317	0.02301	0.03568
*Alistipes*	0.02521	0.01682	0.02556
*Desulfovibrio*	0.01041	0.01960	0.01083
*Helicobacter*	0.01087	0.009667	0.01538
*Ruminococcus*	0.01063	0.01047	0.006793
*Mucispirillum*	0.006149	0.008196	0.004630
*Roseburia*	0.005233	0.009669	0.003542
*Rikenella*	0.009396	0.004176	0.003463
*Odoribacter*	0.005922	0.005754	0.004721
*Coprococcus*	0.003805	0.004305	0.004785
*[Prevotella]*	0.003983	0.003319	0.001441
*Parabacteroides*	0.001715	0.002767	0.001750
*AF12*	0.001829	0.002081	0.001818
*Allobaculum*	0.001037	0.001951	0.001722
*[Ruminococcus]*	0.0009200	0.001982	0.001281
*Dehalobacterium*	0.001366	0.001607	0.001180
*Butyricicoccus*	0.001929	0.0009579	0.001078
*Prevotella*	0.001276	0.001219	0.001337

### Effects of WSQL on serum metabolites

**Figure 5:**
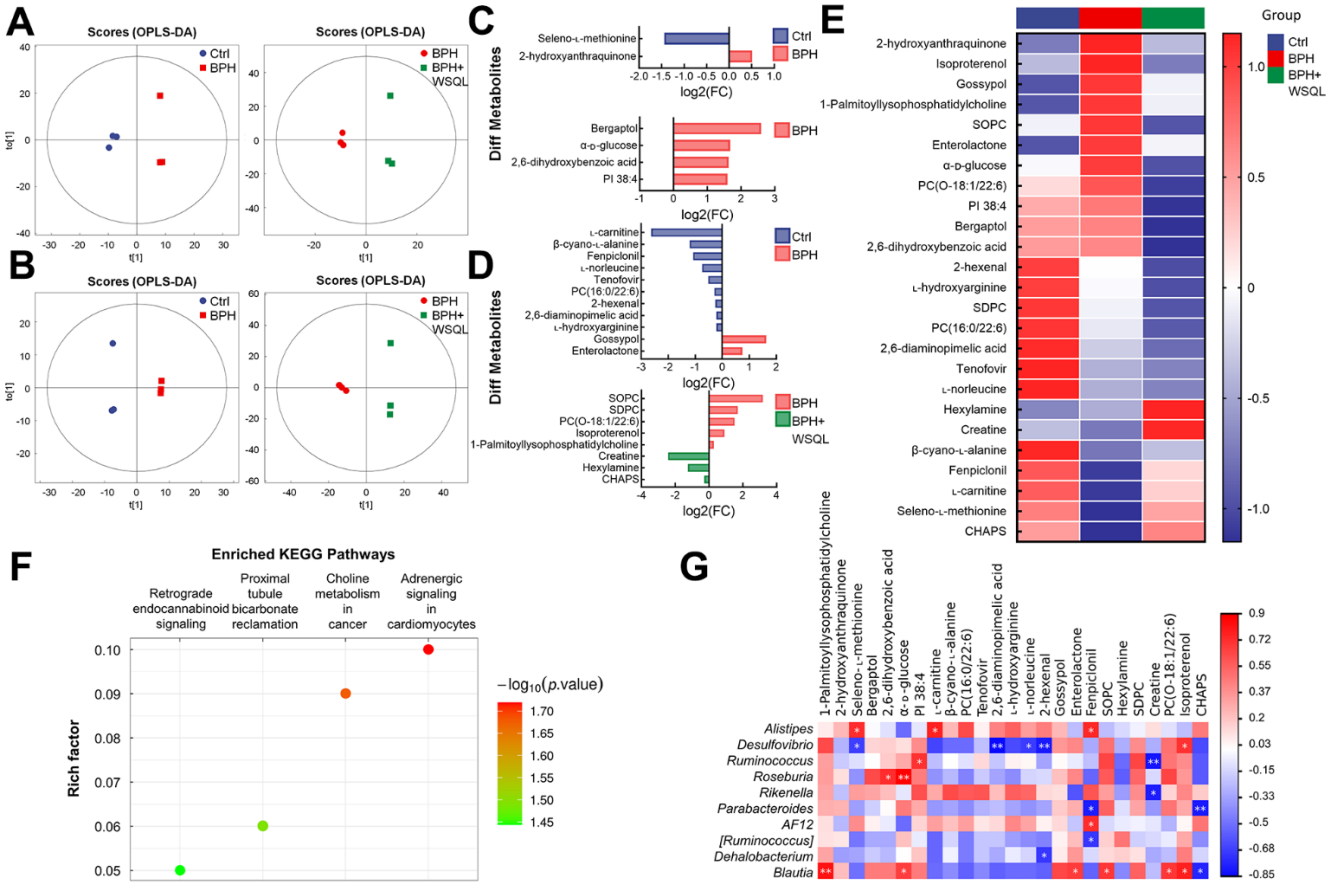
**Analysis for the effect of WSQL treatment on metabolites in TP-induced BPH mice.** OPLS-DA score plots in (**A**) negative ion mode and (**B**) positive ion mode for pair-wise comparisons of BPH with the Ctrl and BPH+WSQL groups respectively. Different metabolites in (**C**) negative ion mode and (**D**) positive ion mode for pair-wise comparisons of BPH with the Ctrl and BPH+WSQL groups respectively. OPLS-DA VIP > 1, *p* < 0.05. (**E**) Heatmap of hierarchical clustering of significantly different metabolites. (**F**) KEGG enrichment pathway bubble diagram between BPH and BPH+WSQL groups. (**G**) Association heatmap between metabolic markers and gut microbial markers. ** *p* < 0.01 and * *p* < 0.05. *n* = 3 for (**A**–**G**).

Drug treatment is usually associated with serum metabolic levels; the dysregulation of the intestinal ecology contributes to the onset of BPH by altering the metabolic profile [17]. Therefore, we analyzed mouse serum using non-targeted metabolomics to determine the effect of WSQL on serum metabolites. The OPLS-DA analysis demonstrated that the metabolic profiles of the BPH group were significantly different from those of the Ctrl and BPH+WSQL groups (Figure 5A, 5B). A total of 25 differential metabolites (OPLS-DA VIP > 1, *p* < 0.05) were obtained from a comparison between the BPH group and both the Ctrl and BPH+WSQL groups (Figure 5C, 5D and Table 3). The mean relative levels of the 25 differential metabolites were calculated for heatmap analysis, of which 2-hydroxyanthraquinone, gossypol, enterolactone, bergaptol, and isoproterenol were observed to be upregulated after TP injection. On the contrary, seleno-L-methionine, L-carnitine, β-cyano-L-alanine, fenpiclonil, and 3-(3-cholamidopropyl) dimethylammonium)-1-propanesulfonate (CHAPS) were downregulated, and a reverse trend for all these metabolites was observed after WSQL administration (Figure 5E). This suggests that these metabolites are related to the role of WSQL in BPH progression. KEGG enrichment pathway analysis between the BPH and BPH+WSQL groups revealed four pathways enriched after WSQL administration, with adrenergic signaling in cardiomyocytes showing the highest significance (Figure 5F). Combining differential metabolites and the top 20 genera of gut microbiota with *Blautia*, a marker genus obtained from LDA for the correlation heatmap (Figure 5G), L-carnitine was observed to be positively correlated with *Alistipes* (*p* < 0.05). *Desulfovibrio* was negatively correlated with seleno-L-methionine and positively correlated with isoproterenol (*p* < 0.05). *Blautia* exhibited a positive correlation with isoproterenol (*p* < 0.05). We hypothesized in this regard that the beneficial effects of WSQL are associated with improved levels of androgenic, antioxidant, and anti-inflammatory related factors.

**Table 3:** Significantly different metabolites (*n* = 3).

**BPH vs. Ctrl**					
**Adduct**	**Name**	**VIP**	***p*-value**	**Mean of Ctrl**	**Mean of BPH**
[M-H]^-^	2-hydroxyanthraquinone	1.8168	0.01204	128994411.90	183589773.00
[M-H]^-^	Seleno-L-methionine	1.7911	0.01303	133993423.30	49240129.86
(M+H)^+^	L-carnitine	1.2972	0.000003267	32964621.02	5316028.99
(M+H-H2O)^+^	β-cyano-L-alanine	1.7766	0.0003670	94974027.87	41087100.91
[M+H]^+^	PC(16:0/22:6)	6.6541	0.007165	4851009095.00	3967203733.33
[M+H]^+^	Tenofovir	1.9212	0.01700	224939704.00	156944398.03
[M+H-CH5O2N]^+^	2,6-diaminopimelic acid	1.2495	0.03070	287616275.17	246961248.63
[M+H-CH5O2N]^+^	L-hydroxyarginine	1.2739	0.03455	345650416.57	298499252.77
(M+H)^+^	L-norleucine	13.3179	0.03557	8853604762.67	5292570278.33
[M+H]^+^	2-hexenal	3.3052	0.03893	1399144313.00	1175751459.33
[M+H-H2O]^+^	Gossypol	2.3994	0.04627	51627276.44	160207638.91
[M+H]^+^	Enterolactone	1.2420	0.04795	42267982.94	70848906.22
[M+H+2i]^+^	Fenpiclonil	3.1473	0.04981	402691302.33	191017470.31
**BPH+WSQL vs. BPH**					
**Adduct**	**Name**	**VIP**	***p*-value**	**Mean of BPH**	**Mean of BPH+WSQL**
[M-H]^-^	Bergaptol	5.0075	0.003567	881403346.33	145551903.27
[M-H]^-^	2,6-dihydroxybenzoic acid	1.7948	0.01089	139669358.27	44993675.45
[M-H]^-^	α-D-glucose	1.0883	0.03036	54670690.26	17129984.86
[M-H]^-^	PI 38:4	2.3228	0.04658	245617630.40	81202515.88
(M-H+2Na)^+^	SOPC	8.8948	0.01211	5389527468.33	590415400.94
[M+H]^+^	Hexylamine	2.1141	0.01560	76806554.85	182320474.50
[M+H]^+^	SDPC	4.1935	0.02559	1530341865.00	470568294.03
[M+H]^+^	Creatine	1.8270	0.02640	19865289.98	106642416.86
[M+H]^+^	PC(O-18:1/22:6)	1.7935	0.03392	295126492.33	102913267.84
[M+H-H2O]^+^	Isoproterenol	1.0148	0.03793	325495570.23	174492378.27
[M+H-C5H13SO3N]^+^	CHAPS	1.0824	0.04800	59587290.81	72499396.99
(2M+Na)+	1-Palmitoyllysophosphatidylcholine	1.8320	0.0137	393662882.70	321620658.77

Adduct: Adduct ion information for compounds; VIP: variable importance for the projection of OPLS-DA; PC(16:0/22:6): 1-palmitoyl-2-docosahexaenoyl-sn-glycero-3-phosphocholine; SOPC: 1-Stearoyl-2-oleoyl-sn-glycerol 3-phosphocholine; SDPC: 1-stearoyl-2-docosahexaenoyl-sn-glycero-3-phosphocholine; PC(O-18:1/22:6): 1-(1z-octadecenyl)-2-(4z,7z,10z,13z,16z,19z-docosahexaenoyl)-sn-glycero-3-phosphocholine; CHAPS: 3-(3-cholamidopropyl) dimethylammonium)-1-propanesulfonate.

### Evaluation of the effects of WSQL on inflammation and oxidative stress-related pathways in BPH mice

**Figure 6:**
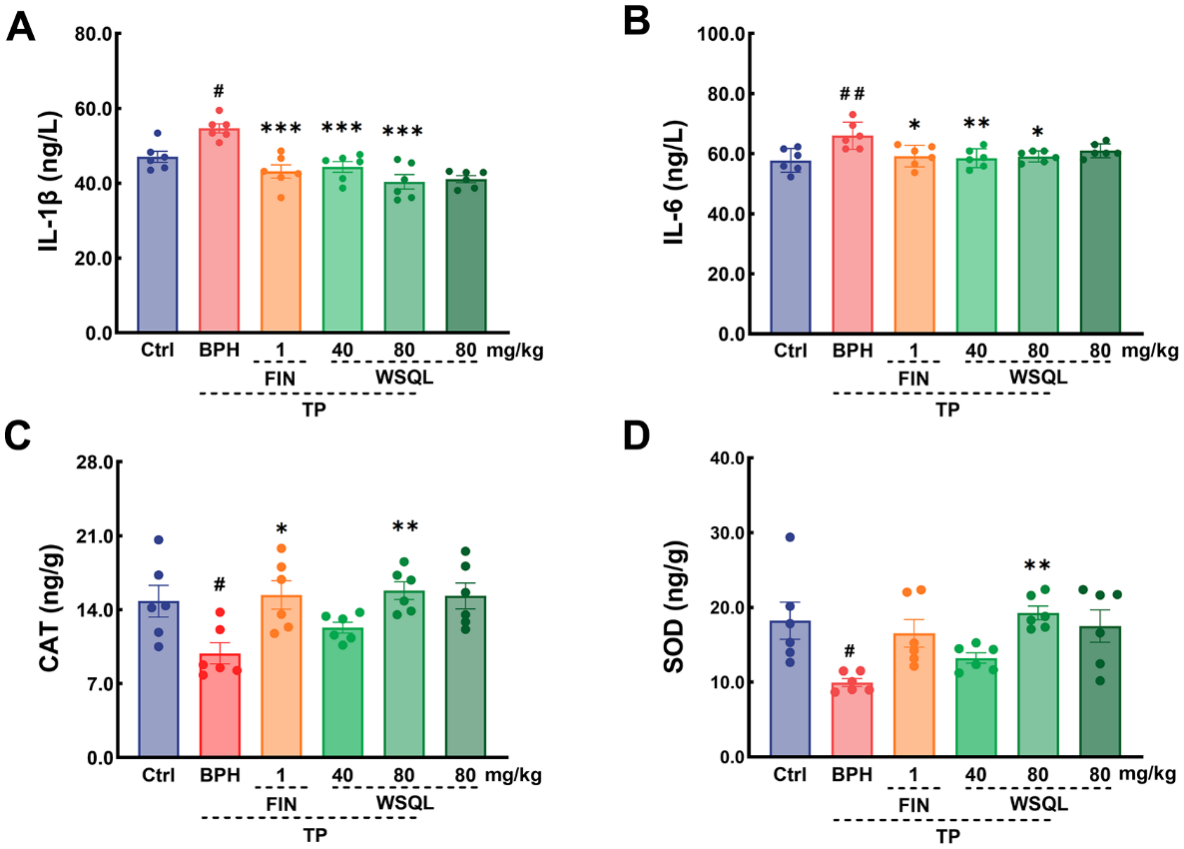
Effect of WSQL on expressions of (**A**) IL-1β and (**B**) IL-6 in serum, and (**C**) CAT, (**D**) SOD in prostate tissue of mice. Data are expressed as means ± SEM. ^##^ *p* < 0.01 and ^#^ *p* < 0.05 vs. Ctrl group, *** *p* < 0.001, ** *p* < 0.01 and * *p* <0.05 vs. BPH group. *n* = 6 for (**A**–**D**).

A few studies have shown that drugs that exert anti-inflammatory and antioxidant effects can have a therapeutic efficacy on BPH [2, 34, 45]. Compared to that in the BPH group, IL-6 and IL-1β levels were significantly lower in the FIN and WSQL administration groups (*p* < 0.05) (Figure 6A, 6B). These results indicated a significant improvement in inflammation levels following WSQL administration.

To determine the effect of WSQL on the antioxidant activity in mouse prostate tissue, we examined the contents of both CAT and SOD (Figure 6C, 6D). After BPH modeling, the contents of CAT and SOD in mouse prostate tissues were significantly lower (*p* < 0.05), and FIN exerted a significant elevating effect on CAT content (*p* < 0.05). Compared to the BPH group, the BPH+WSQL (80 mg/kg) group showed significantly higher CAT and SOD contents, which were lower after modeling (*p* < 0.01).

**Figure 7:**
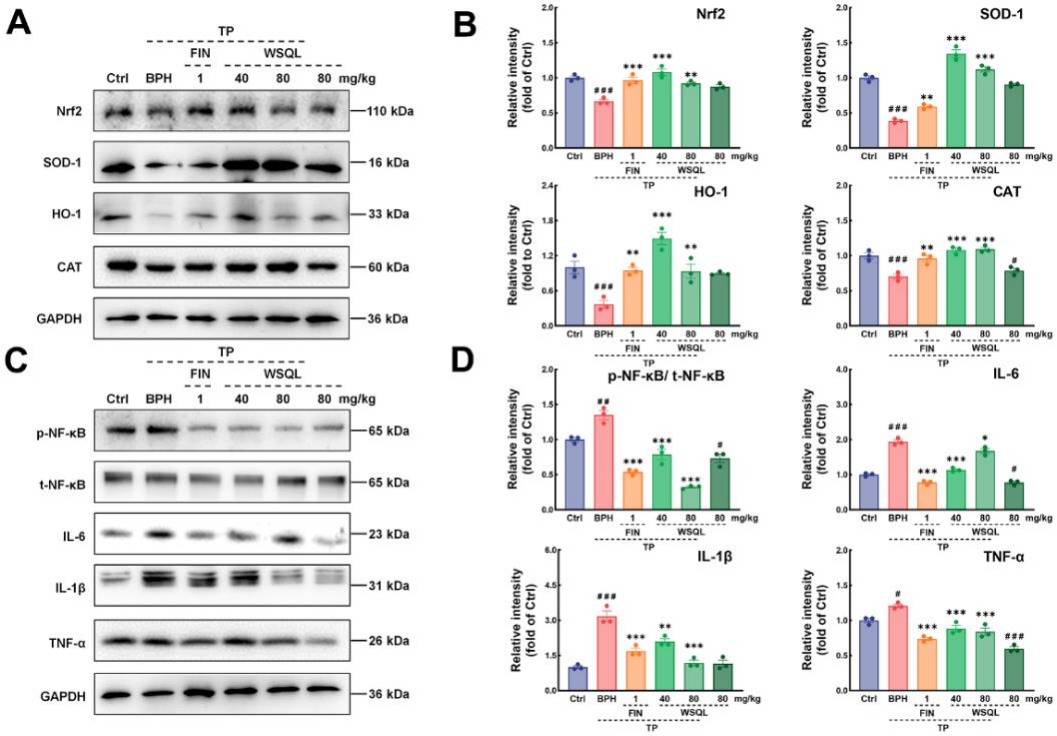
**Effects of WSQL on the Nrf2 and NF-κB signaling pathways in the BPH model mice.** (**A**) The protein levels and (**B**) quantitative analyses of Nrf2, SOD-1, HO-1 and CAT as well as (**C**) the protein levels and (**D**) quantitative analyses of p-NF-κB, t-NF-κB, IL-6, IL-1β and TNF-α were determined using western blotting. GAPDH or the corresponding total protein (t-NF-κB) was used as an internal control and were reported as the fold change of the Ctrl group. The data are presented as the mean ± SEM. ^###^ *p* < 0.001 and ^#^ *p* < 0.05 vs. Ctrl group, *** *p* < 0.001, ** *p* < 0.01 and * *p* < 0.05 vs. BPH group. *n* = 3 for (**A**–**D**).

To further elucidate the molecular mechanism underlying the WSQL’s antioxidant activity, we studied the Nrf2 pathway, which plays a key role in coordinating cellular antioxidant defenses and maintaining cellular redox homeostasis. Western blotting showed a significant reversal of the decreased Nrf2 levels in the BPH group following FIN and WSQL administration (*p* < 0.01) (Figure 7A, 7B). Additionally, the expression of HO-1 and SOD-1 is regulated by Nrf2, both of which are critical in maintaining antioxidant/oxidant homeostasis [2]. CAT is another Nrf2-activated antioxidant signaling pathway-related factor. Consistently, significant increases in the contents of SOD-1, HO-1, and CAT were observed in the BPH+FIN and BPH+WSQL groups compared with those in the BPH group (*p* < 0.05).

As shown in Figure 7C, 7D, the expression of NF-κB was examined to further investigate the mechanism of action of WSQL in suppressing inflammation. The results showed that the expression of NF-κB in the BPH group was higher after TP injection compared with the Ctrl group (*p* < 0.001). TP injection significantly increased the expression of inflammatory markers such as IL-6, IL-1β, and TNF-α (*p* < 0.05). After FIN and WSQL administration, the expression of NF-κB and downstream inflammatory markers was significantly lower (*p* < 0.05).

## DISCUSSION

BPH is a non-malignant prostate enlargement that can lead to lower urinary tract symptoms [2]. Given the risks associated with surgery and the side effects of currently approved drug treatments [46, 47], there is an urgent need for safe and effective treatment methods. In this study, we evaluated the effects of WSQL on the prostate in a TP-induced BPH model in BALB/c mice using histological analysis, gut microbiomics, serum non-targeted metabolomics, and biochemical methods. The experimental results revealed that the alleviating effect of WSQL on BPH was correlated with its anti-inflammatory and antioxidant properties.

BPH leads to symptoms of transition zone hyperplasia, prostate inflammation and elevated prostate weight and index. The development and progression of prostate diseases, including BPH, are often accompanied by changes in androgen levels. Moreover, various interventions improve BPH function by normalizing androgen levels [1, 2]. TGF-α is a growth factor that can stimulate prostate growth, and its expression is believed to be regulated by androgens [48]. After TP injection, the levels of PSA, androgens, and TGF-α increased, which is consistent with findings from previous studies [2, 42, 49]. After WSQL administration, symptoms related to the prostate and altered levels of androgen and TGF-α caused by BPH improved. Additionally, WSQL exerted minimal effect on body weight, liver, spleen and heart indexes and their histopathology in mice, indicationg its safety. These results support our working hypothesis that WSQL attenuates BPH symptoms.

Li et al. [17] showed that BPH is associated with altered abundance and diversity of gut microbiota and intestinal metabolomics in rats. However, the causal relationship between gut microbiota and BPH is unclear. Dong et al. [50] reported that fecal microbiota transplantation alleviated the enlarged prostate volume in a mice colitis model, suggesting the importance of gut microbiota for BPH. Modifying the gut microbiota can indirectly affect prostate health by activating the immune system and producing proinflammatory cytokines [13]. Considering the important role of microbiome homeostasis in maintaining host health, we detected significant differences in the microbial community structure and function following TP injection and WSQL treatment. After TP injection, WSQL treatment resulted in increased and decreased abundance of four and eight bacterial genera, respectively, suggesting a regulatory effect of WSQL on the gut microbiota. Gut microbiota abundance and metabolism interact with sex hormone levels, including androgen [51]. T is one of the substrates of CYP3A4, and metabolism of xenobiotics by CYP450 in the prediction of pathways associated with gut microbiota may respond to this potential role in the BPH disease process. There are studies demonstrate that serum T positively regulates the abundance of *Roseburia*, *AF12*, *Allobaculum* and *Parabacteroides* [52–54], as illustrated by our results. In another study, T was negatively correlated with *Alistipes* abundance [55], which is consistent with the relationship between T level and *Alistipes* abundance in the BPH and WSQL administration groups in this experiment. Additionally, the gut microbiota has a similarly complex interrelationship with inflammation. A comparison of the taxonomic composition analysis plots at the phylum level of the three groups revealed a change in the decline of Firmicutes/Bacteroidetes, which is considered as a marker of malnutrition and is associated with inflammatory bowel disease [56]. At the genus level, *Blautia* and *Coprobacillus*, which were considered as BPH marker genera in the present study, and *Parabacteroides* were positively associated with the levels of inflammatory factors such as TNF-α [43, 57–59]. Enriching *Desulfovibrio* in the BPH group may cause inflammation by damaging the intestinal barrier by producing excessive H_2_S [60]. On the contrary, *Alistipes*, *Prevotella*, and *Butyricicoccus* are considered beneficial microorganisms associated with increased production of short chain fatty acids [44, 61, 62], thus affecting the integrity of the intestinal barrier, inhibiting the secretion of pro-inflammatory factors, and stimulating anti-inflammatory factors. Their abundance increased after WSQL administration compared to that in the BPH group. Treatment of BPH mice with WSQL resulted in improvement of the aforementioned inflammation-related gut microbiota, suggesting that WSQL alleviates BPH symptoms by improving the gut microbiota. One of the mechanisms of this mitigating effect may be the inhibition of inflammation-related pathways. Microbial relevant metabolic pathway analysis with significant differences indicated that TP injection upregulated three pathways. WSQL appears to act therapeutically on BPH by upregulating and downregulating the beneficial and harmful bacteria in the gut microbiota of BPH, respectively.

A gut-genitourinary axis is involved in regulating intestinal microbiota by BPH, which regulates the metabolic activity of the body [17]. Therefore, we performed a serum non-targeted metabolomics analysis to test the hypothesis of a possible association between gut microbiota and serum metabolomics and the effect of WSQL on the progression of BPH. Twenty-four significantly differentially expressed metabolites were characterized in positive and negative ion patterns. In prostatic hyperplasia, WSQL may affect inflammatory levels and exhibit antioxidant capacity by regulating metabolites. L-Carnitine activates Nrf2 and plays a crucial role in the endogenous metabolism of short-chain fatty acids by the gut microbiota, which controls different biological processes such as systemic inflammation [63]. Seleno-L-methionine has antioxidant and anti-inflammatory effects, probably by inducing antioxidant proteins and glutathione peroxidase [64]. Seleno-L-methionine attenuates inflammation via the activated selenoprotein S-mediated Toll-like receptor 4/NF-κB signaling pathway [65]. These metabolites were all higher in the BPH+WSQL group than in the BPH group, suggesting the antioxidant and anti-inflammatory capacity of WSQL. Bergaptol is an inhibitor of CYP3A4 (a type of CYP450) [66], which is involved in drug metabolism. T is one of the substrates of CYP3A4, and its metabolism in the BPH+WSQL group could have increased due to a decline in bergaptol. Isoproterenol pronounces the production of inflammatory factors and impairs the activity of antioxidant enzymes [67, 68]. Furthermore, isoproterenol directly effects on the prostate, which may be independent of T levels [69]. This effect is manifested by the marked enlargement of the gland, an increase in the volume of epithelial cells and lumen, and an increase in the average diameter of the lumen of glandular vesicles. Reversal of a range of serum metabolite levels during WSQL administration to BPH model mice suggests that WSQL may play a role in increasing antioxidant and anti-inflammatory factor levels. The significantly changed metabolites selected showed significant correlation with gut microbes (*p* < 0.05), which indicates that there may be metabolite-gut microbial interactions during the dosing action of WSQL in this study. Further experimental verification of these effects was performed in this study.

The proliferation of prostate cells in BPH further develops under chronic inflammation, causing these cells to undergo a cycle of damage and repair over time. Inflammatory factors are chronically overexpressed under BPH conditions, producing cytokines including and not limited to, IL-1β and IL-6. These inflammatory factors often affect affecting abnormal prostate proliferation [1, 39, 70]. Consistently, the data from our study indicated the upregulation of these cytokines after TP injection. FIN and WSQL treatment successfully reduced the TP-stimulated upregulation of these inflammatory cytokines in both the serum and prostate. Moreover, our study supports the hypothesis that inflammation mediates the progression of BPH.

Oxidative stress-mediated and -induced lipid peroxidation and cell proliferation can significantly affect the normal physiological state of blood vessels and induce cell mutation, proliferation, and differentiation. Nrf2 is involved in BPH pathogenesis and may be a target for drug interventions [6]. The Nrf2 pathway plays a role in oxidant/antioxidant homeostasis and inflammation [71]. Nrf2 enters the nucleus and binds to antioxidant response elements, leading to higher expression of several downstream genes, including *HO-1*. Nrf2 and HO-1 can inhibit the nuclear translocation of NF-κB [72]. NF-κB activates the expression of genes encoding catabolic enzymes, inflammatory mediators, and cytokines. The injection of T in the BPH model induces excessive inflammation and weakens cellular antioxidant mechanisms [1], which may explain the lower CAT and SOD contents in prostate cells in the BPH group compared with those in the Ctrl group. This decrease in antioxidant capacity was alleviated to varying degrees by the administration of FIN and WSQL. Western blotting confirmed that WSQL attenuated BPH symptoms via upregulating the Nrf2/HO-1 pathway and downregulating NF-κB and related inflammatory factors.

## CONCLUSIONS

WSQL treatment significantly reduced prostate hyperplasia, improved the histopathological characteristics of prostate tissues and regressed serum hormone levels. The therapeutic effect of WSQL on BPH was associated with an altered abundance and diversity of gut microbiota and serum metabolomics in mice. Additionally, the antiproliferative effect of WSQL was attributed to its potent anti-inflammatory and antioxidant effects. Our experiments primarily focused on preventing BPH symptoms through antioxidant and inflammation-reducing effects. Exploring the mechanisms revealed that WSQL protected against TP-induced BPH via modulation of, at least partly, Nrf2 and NF-κB pathway. Our study demonstrated the potential use of WSQL in BPH therapy and other types of BPH research and supported that the modulation of Nrf2 and NF-κB pathways has favorable therapeutic effects in improving BPH. More in-depth studies on the specific gut microbiota or serum metabolites that play a role in the mitigating of BPH by WSQL are still needed.
